# Foveal Hypoplasia in a Child With Tyrosinase-Positive Albinism

**DOI:** 10.7759/cureus.44558

**Published:** 2023-09-02

**Authors:** Alexandra Kavalaraki, Konstantinos Paraskevopoulos, Marianna Kavalaraki, Christina Karakosta, Maria Liaskou

**Affiliations:** 1 Ophthalmology, Penteli General Children's Hospital, Athens, GRC; 2 Ophthalmology, Athens Naval Hospital, Athens, GRC; 3 Ophthalmology, Korgialenio-Benakio Hellenic Red Cross Hospital, Athens, GRC

**Keywords:** foveal hypoplasia, tyrosinase-positive albinism, oculocutaneous albinism (oca), oca2, iris transillumination, nystagmus, pendular nystagmus, oct (optical coherence tomography)

## Abstract

The purpose of this article is to report a case of bilateral foveal hypoplasia in an eight-year-old girl who presented to the ophthalmology department due to poor vision in both eyes. Clinical examination revealed bilateral nystagmus, decreased vision, as well as iris transillumination. Dilated fundus examination indicated the absence of light reflex around the foveal area and optical coherence tomography (OCT) imaging exhibited the absence of the fovea centralis depression. These findings, in addition to the patient's light-colored hair and skin complexion, raised suspicion for albinism. The patient was referred for genetic testing and the results confirmed the diagnosis of tyrosinase-positive oculocutaneous albinism (OCA2).

## Introduction

Foveal hypoplasia (FH) refers to a retinal abnormality characterized by the insufficient development or complete absence of the fovea centralis in the macula lutea. The foveal pit formation normally begins during the 25th week of fetal development and is concluded 15-45 months postpartum [1]. This process involves the drifting of the inner retinal layers peripherally and the central concentration of cone photoreceptors in a dense manner. The cones eventually form a multicellular layer and their outer segments (OS) elongate, which is characterized as cone specialization and is of critical importance for good visual acuity (VA) [2]. As a result, the fovea comprises cones and Müller cells, whereas rod cells are absent. Normal foveal development highly depends on the presence of the foveal avascular zone (FAZ) [3].

Disturbance of the foveal developmental process results in FH, an abnormality that is associated with a number of ocular as well as systematic diseases, including albinism, aniridia, achromatopsia, retinopathy of prematurity, familial exudative vitreoretinopathy, optic nerve hypoplasia, incontinentia pigmenti, congenital retinal macrovessel, and Stickler syndrome. Genetic factors along with risk factors such as prematurity contribute to the pathogenesis of foveal developmental disruption [2]. FH can also present as an isolated finding, most commonly in cases where specific genes such as PAX6 and SLC38A8 were found to be involved [4]. Clinical manifestations of FH typically include low VA and nystagmus [5].

Optical coherence tomography (OCT) along with OCT-angiography (OCT-A) and fluorescein angiography are used for the diagnosis of FH. OCT demonstrates a lack of the foveal pit as well as the persistence of the inner retinal layers, whereas OCT-A and fluorescein angiography exhibit the absence of the FAZ, which is also a common finding in FH [6]. A grading system of FH has been proposed by Thomas et al., based on structural features using a spectral-domain OCT [7]. The Leicester Grading system describes four grades of typical FH; Grade 1 is characterized by a depthless foveal pit and incursion of the plexiform layers, Grade 2 describes the same morphology but lacks a pit depression, Grade 3 additionally lacks the OS elongation, and Grade 4 is furthermore identified by the absence of the outer nuclear layer (ONL) widening. Therefore, the deficiency of the plexiform extrusion is a critical structural feature for the diagnosis of FH. A grade of atypical FH has also been described and concerns photoreceptor degeneration. The association between different grades of FH and VA was also studied by Thomas et al., and it was determined that Grade 1 was correlated to the best VA, whereas Grades 2-4 were marked by a gradually lower VA. This was attributed to the fact that Grades 1 and 2 are defined by the presence of cone specialization, while Grades 3 and 4 lack OS lengthening.

The aim of this article is to present a case of FH in an eight-year-old girl who presented to our ophthalmology department with poor vision, nystagmus, and peripheral iris transillumination.

## Case presentation

An eight-year-old girl presented to our department with a complaint of poor vision in both eyes since birth. On clinical examination, we noticed the presence of very light-colored hair and fair skin, as well as light blue-colored irises. Best corrected visual acuity (BCVA) was measured to be 0.4 and 0.5 for the right (OD) and left eye (OS) respectively (decimal). Cycloplegic refraction was +6.50 DS + 0.50 DC X 90 in the OD and + 6.50 DS + 0.50 X 110 in the OS. The child had an eyeglasses prescription of + 5.75 DS OU. Furthermore, a latent pendular nystagmus characterized by horizontal as well as torsional oscillations was detected in both eyes. A slit lamp examination revealed a mild peripheral iris transillumination, which was more prominent in the right eye. The patient reported mild photophobia. The anterior chambers were normal in both eyes. Testing with Ishihara pseudoisochromatic plates revealed no color vision defect.

Previous ophthalmic history revealed an accommodative esotropia of 45 prism diopters of the OD, which was first diagnosed in our department at the age of six months. However, further data was missing due to the loss of follow-up. The patient had no previous history of major illnesses and there was no history of prematurity. Family history was insignificant.

**Figure 1:**
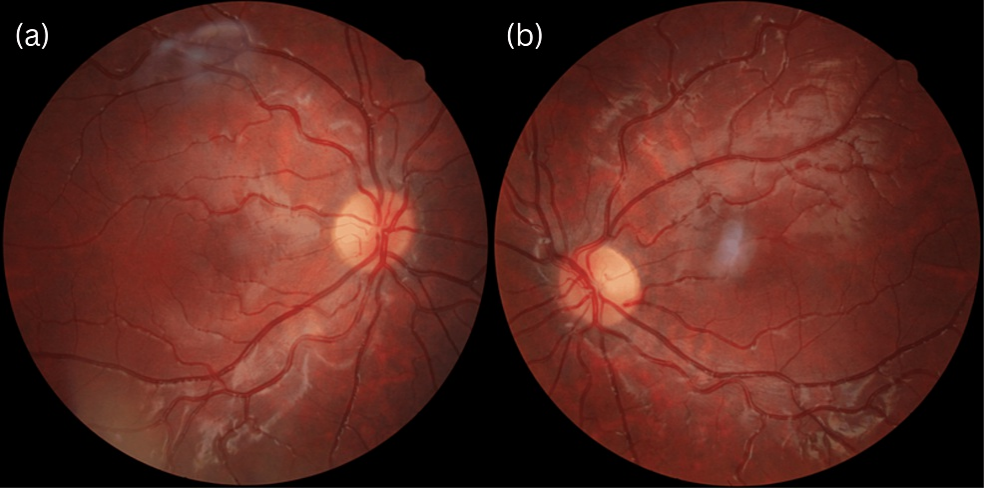
Fundus photography of the OD (a) and OS (b) Reduction of the circumfoveal light reflex is notable in both eyes. There is no disturbance of macular pigmentation and the choroidal vessels are not visible. OD: Oculus dexter, OS: Oculus sinister

**Figure 2:**
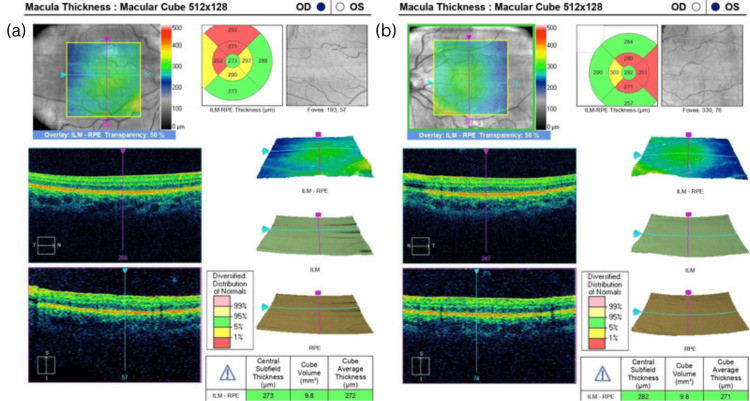
OCT images of the right eye (a) and left eye (b) Absence of foveal pit depression in both eyes. Retinal layers are normally demonstrated. OCT: optical coherence tomography

Dilated fundus examination showed a reduced circumfoveal light reflex in both eyes, which was also confirmed by Fundus photography (Figure 1). However, there were no changes in foveal pigmentation and no abnormalities of the optic discs or vessels were detected. OCT imaging revealed a lack of foveal depression and persistence of the inner retinal layers in both eyes (Figure 2). Nevertheless, the individual retinal layers maintained normal structural characteristics. According to the Leicester grading system [7], this is a case of FH grade 4. RNFL (retinal nerve fiber layer) analysis showed no pathological changes in the peripapillary thickness.

The patient was further referred for genetic testing due to high suspicion of albinism, as a result of the specific phenotype in addition to the clinical and imaging findings. Genetic testing confirmed a mutation in the OCA2 gene, which is the gene responsible for tyrosinase-positive oculocutaneous albinism (OCA type 2).

The patient and her parents consented in writing to participation in a study, in the course of which the images included in this report were taken. This report does not contain any identifying information. This case was reported according to the World Medical Association Declaration of Helsinki.

## Discussion

Albinism refers to a genetically determined group of heterogeneous disorders, which are characterized by melanin synthesis disturbance. Two main categories are recognized: oculocutaneous albinism (OCA), in which the skin, hair, and eyes are affected, and ocular albinism (OA), which affects the eyes only and is X-linked inherited. Moreover, there are several syndromic types of albinism, such as Chediak-Higashi and Hermansky-Pudlak [8]. OCA is an autosomal recessive disorder and can be characterized as either tyrosinase-positive or tyrosinase-negative, which is determined by the hair bulb incubation test [9]. OCA2 results in the synthesis of various amounts of melanin and therefore, skin and hair color can slightly darken throughout the years. OCA2 is caused by mutations of the OCA2 gene on chromosome 15ql2-ql3. On the contrary, tyrosinase-negative albinism is marked by the complete absence of melanin synthesis resulting in white hair and skin, accompanied by more severe ocular manifestations [8]. Until recently, four genes had been found to be responsible for OCA and one gene for OA; however, Montoliu et al. identified seven types of human OCA (OCA 1-7) and six genes responsible for them [10].

Ocular manifestations of albinism include impaired vision, color vision impairment, FH, nystagmus, iris transillumination due to iris hypopigmentation, retinal hypopigmentation, high refractive errors, strabismus, and chiasmal misrouting [8]. Even though albinism has a heterogeneous phenotype and clinical manifestations can vary, FH is considered to be a common feature among different types [11]. A cohort study including 907 patients demonstrated that albinism was the most common genetic cause of typical FH (67.5%) [12]. It is believed that FH is the main cause of low vision in patients with albinism, along with iris transillumination and macular transparency. By applying OCT imaging in 13 patients with albinism, Seo et al. exhibited that FH grading acts as a better predictive value for VA in comparison to iris transillumination and grading of macular transparency [13]. Therefore, it is important to conduct OCT imaging in all patients with low vision and nystagmus.

Similar cases of FH in patients with albinism have been reported in the literature. Chong et al. performed standard and handheld spectral-domain OCT (SD-OCT) imaging in seven pediatric patients with OA or suspected OA, in one patient with OCA and Hermansky-Pudlak syndrome, as well as four control subjects [14]. Abnormalities in foveal architecture, including persistence of the inner retinal layers across the fovea and loss of the thickened photoreceptor nuclear layer, were observed in the first two groups, whereas normal foveal depression was noted in the control group. McCafferty et al. also used SD-OCT imaging for evaluation of the foveal structure in 14 children with albinism and demonstrated that rudimentary pit formation and normal inner and outer segment elongation were associated with a better VA [11]. Our patient presented with numerous clinical characteristics of albinism and OCT imaging further contributed to the clinical evaluation and diagnosis, which was then confirmed as OCA2 with genetic testing.

## Conclusions

FH is a retinal disorder characterized by insufficient development or absence of the fovea centralis and is associated with many ocular and systemic disorders. It is typically accompanied by decreased vision and nystagmus; nevertheless, VA can vary depending on the grade of FH. It is important that OCT imaging be performed in all patients who present with decreased vision and nystagmus, both for diagnostic purposes as well as a predictive value of VA. FH is a common feature in patients with albinism and OCT imaging is critical for the evaluation of the foveal morphology and clinical assessment of the patient. Genetic testing is essential in establishing the diagnosis of albinism and may play a key role in the development of gene therapies in the future.
